# Dataset including whole blood gene expression profiles and matched leukocyte counts with utility for benchmarking cellular deconvolution pipelines

**DOI:** 10.1186/s12863-024-01223-z

**Published:** 2024-05-07

**Authors:** Grant C. O’Connell

**Affiliations:** 1 Molecular Biomarker Core, Case Western Reserve University, Cleveland, OH USA; 2 School of Nursing, Case Western Reserve University, 10900 Euclid Avenue, Cleveland, OH 44106-4904 USA

**Keywords:** RNA-seq, Blood, Methods, Reference dataset, Benchmarking, Deconvolution, White blood cells

## Abstract

**Objectives:**

Cellular deconvolution is a valuable computational process that can infer the cellular composition of heterogeneous tissue samples from bulk RNA-sequencing data. Benchmark testing is a crucial step in the development and evaluation of new cellular deconvolution algorithms, and also plays a key role in the process of building and optimizing deconvolution pipelines for specific experimental applications. However, few in vivo benchmarking datasets exist, particularly for whole blood, which is the single most profiled human tissue. Here, we describe a unique dataset containing whole blood gene expression profiles and matched circulating leukocyte counts from a large cohort of human donors with utility for benchmarking cellular deconvolution pipelines.

**Data description:**

To produce this dataset, venous whole blood was sampled from 138 total donors recruited at an academic medical center. Genome-wide expression profiling was subsequently performed via next-generation RNA sequencing, and white blood cell differentials were collected in parallel using flow cytometry. The resultant final dataset contains donor-level expression data for over 45,000 protein coding and non-protein coding genes, as well as matched neutrophil, lymphocyte, monocyte, and eosinophil counts.

## Objectives

Cellular deconvolution is a computational process that can infer the cellular composition of heterogeneous tissue samples from bulk RNA-sequencing data; it is being increasingly used to help researchers track cell population dynamics, as well as better explore nuclear transcriptional state by controlling for confounding interspecimen differences in cellularity. Well over 50 different algorithms or mathematical approaches been developed for deconvolution [1], and new ones are being proposed regularly.

Generally, these different approaches can have advantages and disadvantages and offer varying levels of performance depending on considerations unique to the specific experimental context, such as the cellular complexity of the tissue under investigation and the desired informational output. In addition to selection of an algorithm, numerous other decisions need to be made when building a deconvolution pipeline for a specific experimental application that can influence accuracy, including the choice of reference gene expression profiles or marker genes, and how to normalize or pre-process the bulk gene expression data [2–4]. In order to ensure optimal performance, ideally, these parameters are selected empirically via benchmark testing, which is typically performed using gene expression data from either in vivo, in vitro, or in silico samples comprised of a known mixture of cell types [5]. While it can be argued that the use of in vivo benchmarking datasets represents the gold standard, few in vivo benchmarking datasets exist, particularly for whole blood, which is the single most profiled tissue in human transcriptomic investigations [6].

As part of a larger study which aimed to assess the drivers of peripheral blood gene expression patterns [7], our group recently used a combination of next generation RNA sequencing and flow cytometry to generate a unique dataset containing whole blood gene expression profiles and matched leukocyte counts from a large cohort of human donors. Given the current lack of in vivo benchmarking datasets that exist for whole blood, these data have value for secondary use in evaluating cellular deconvolution pipelines.

## Data description

To generate the dataset, parallel venous whole blood specimens were collected from 138 adult donors via K_2_EDTA and PAXgene vacutainers at admission to the Emergency Department at Dell-Seton Medical Center (Austin, TX) as described by our group previously [7]. K_2_EDTA vacutainers were used immediately for flow cytometry analysis, while PAXgene vacutainers were stored until downstream RNA isolation.

White blood cell differential was assessed in EDTA-treated whole blood via four angle optical flow cytometry. Relative neutrophil, lymphocyte, monocyte, and eosinophil counts were generated by dividing the absolute counts of the aforementioned leukocyte subpopulations by the absolute total leukocyte count. The final cell counts display a high degree of inter-sample heterogeneity in terms of overall leukocyte composition, and in the case of each cell type, the relative counts span well beyond the adult human reference range (Supplemental Fig. 1) [8], collectively suggesting that the final dataset captures adequate variance in cell counts to be generalizable for use in deconvolution benchmarking.

Total RNA was isolated from PAXgene stabilized whole blood using spin column-based solid phase extraction. RNA purity and integrity were assessed using a combination of spectrophotometry and chip capillary electrophoresis. Ribosomal RNA and globin mRNA-depleted cDNA libraries were prepared and subsequently sequenced via illumina sequencing using paired-end 150 bp reads. Reads were aligned to human reference genome GRCh38 and the counts of mapped were reads summarized at the gene level. On average, approximately 40 million reads were generated per sample, and greater than 90% of reads map to the reference genome (Supplemental Fig. 2) [9]. Transcript from a total of 45,429 genes was detected, including a median of 15,425 protein-coding genes, 4,822 lncRNA associated genes, 2,947 pseudogenes, and 196 miRNA associated genes per sample (Supplemental Fig. 3) [10]. This suggests that the final dataset contains adequate genomic coverage to be compatible with a wide range of reference gene expression profiles and marker gene lists that could be employed in cellular deconvolution benchmarking tests.

Importantly, deconvolution of the final gene expression data via marker genes using a simple principal components analysis-based approach [11] yields inferred cell counts that are highly correlated with the actual cell counts measured with flow cytometry (Spearman’s rho = 0.73–0.84; Supplemental Fig. 4) [12], indicating that the final gene expression data and flow cytometry data are correctly integrated terms of donor-level matching, and that the dataset has true utility for this particular secondary use.

All final data are available from the National Center for Biotechnology Information (NCBI) Sequence Read Archive (SRA) via permanent accession number SRP429744 [13]. Raw sequencing data are available as .fastq files and can be downloaded individually or in bulk using the SRA run selector. Quality metrics associated with the source RNA, as well as basic demographic information and white blood cell counts for all donors are available via the attribute slots of linked BioSample records. RNA quality metrics include RNA integrity numbers, 260:230 ratios, and 260:280 ratios, donor demographic information includes age, sex, race, and ethnicity, while white blood cell counts include relative neutrophil, lymphocyte, monocyte, and eosinophil counts, all under accordingly named attribute slots. All cell counts are listed as decimal formatted proportions. These linked BioSample attributes can be bulk downloaded as metadata using the SRA run selector when downloading sequencing data.

**Table 1:** Overview of files and datasets

	Name of data file/data set	File types (file extension)	Data repository and identifier (DOI or accession number)
Dataset 1	Raw sequencing data and linked metadata including leukocyte counts	Raw read counts (.fastq); Sample metadata (.txt)	NCBI Sequence Read Archive (Accession number: SRP429744, https://identifiers.org/ncbi/insdc.sra:SRP429744) [13]
Data file 1	Supplemental Fig. 1	Figure (.pdf)	figshare (DOI: 10.6084/m9.figshare.25155521) [8]
Data file 2	Supplemental Fig. 2	Figure (.pdf)	figshare (DOI: 10.6084/m9.figshare.25155566) [9]
Data file 3	Supplemental Fig. 3	Figure (.pdf)	figshare (DOI: 10.6084/m9.figshare.25155569) [10]
Data file 4	Supplemental Fig. 4	Figure (.pdf)	figshare (DOI: 10.6084/m9.figshare.25155572) [12]

## Limitations

Like any dataset, there are caveats and limitations that should be considered when planning for future use. Perhaps most notably, it is important to consider that the dataset only contains cell count data for the four most abundant circulating white blood cell populations, as opposed to more granular cell populations which can be more extensively quantified via fluorescent flow cytometry. With respect to future use for benchmarking cellular deconvolution pipelines, this may make the dataset best suited to evaluate the performance of marker-based and reference-based deconvolution approaches such as CIBERSORT [14], xCell [15], and DeMix [16], as the cell types for which counts are to be inferred are dictated a priori by the user. However, even in the instance of reference-free deconvolution approaches, the dataset could still be employed to assess how well the collective inferred counts of any more granular cell types that are output correlate with actual cell counts of the parent cell population.

## Data Availability

The data described in this Data Note can be freely and openly accessed from the National Center for Biotechnology Information (NCBI) Sequence Read Archive (SRA) via permanent accession number SRP429744; please see Table 1 and reference [13] for details and links to the data. All Supplemental Figures are available via figshare; please see Table 1 and references [8–10, 12] for details and links to these materials.

## References

[1] Avila Cobos F, Vandesompele J, Mestdagh P, De Preter K (2018). Computational deconvolution of transcriptomics data from mixed cell populations. Bioinformatics.

[2] Avila Cobos F, Alquicira-Hernandez J, Powell JE (2020). Benchmarking of cell type deconvolution pipelines for transcriptomics data. Nat Commun.

[3] Newman AM, Gentles AJ, Liu CL (2017). Data normalization considerations for digital tumor dissection. Genome Biol.

[4] Sutton GJ, Poppe D, Simmons RK (2022). Comprehensive evaluation of deconvolution methods for human brain gene expression. Nat Commun.

[5] Jin H, Liu Z (2021). A benchmark for RNA-seq deconvolution analysis under dynamic testing environments. Genome Biol.

[6] Giles CB, Brown CA, Ripperger M (2017). ALE: automated label extraction from GEO metadata. BMC Bioinformatics.

[7] O’Connell GC, Wang J, Smothers C (2023). Donor white blood cell differential is the single largest determinant of whole blood gene expression patterns. Genomics.

[8] O’Connell GC. Supplemental Figure 1. figshare. 2024. 10.6084/m9.figshare.25155521.

[9] O’Connell GC. Supplemental Figure 2. figshare. 2024. 10.6084/m9.figshare.25155566.

[10] O’Connell GC. Supplemental Figure 3. figshare. 2024. 10.6084/m9.figshare.25155569.

[11] O’Connell GC (2023). Variability in donor leukocyte counts confound the use of common RNA sequencing data normalization strategies in transcriptomic biomarker studies performed with whole blood. Sci Rep.

[12] O’Connell GC. Supplemental Figure 4. figshare. 2024. 10.6084/m9.figshare.25155572.

[13] (2023) Whole blood gene expression data and matched white blood cell counts generated from human donors diagnosed with a variety of chronic diseases. NCBI Sequence Read Archive. https://identifiers.org/ncbi/insdc.sra:SRP429744.

[14] Newman AM, Liu CL, Green MR (2015). Robust enumeration of cell subsets from tissue expression profiles. Nat Methods.

[15] Aran D, Hu Z, Butte AJ (2017). xCell: digitally portraying the tissue cellular heterogeneity landscape. Genome Biol.

[16] Ahn J, Yuan Y, Parmigiani G (2013). DeMix: deconvolution for mixed cancer transcriptomes using raw measured data. Bioinformatics.

